# Multi-center real-world comparison of the fully automated Idylla™ microsatellite instability assay with routine molecular methods and immunohistochemistry on formalin-fixed paraffin-embedded tissue of colorectal cancer

**DOI:** 10.1007/s00428-020-02962-x

**Published:** 2020-11-10

**Authors:** Ana Velasco, Fatma Tokat, Jesper Bonde, Nicola Trim, Elisabeth Bauer, Adam Meeney, Wendy de Leng, George Chong, Véronique Dalstein, Lorand L. Kis, Jon A. Lorentzen, Snjezana Tomić, Keeley Thwaites, Martina Putzová, Astrid Birnbaum, Romena Qazi, Vanessa Primmer, Barbara Dockhorn-Dworniczak, Javier Hernández-Losa, Fernando A. Soares, Asaf A. Gertler, Michal Kalman, Chris Wong, Dirce M. Carraro, Ana C. Sousa, Rui M. Reis, Stephen B. Fox, Matteo Fassan, Marie Brevet, Sabine Merkelbach-Bruse, Richard Colling, Elizabeth Soilleux, Ryan Yee Wei Teo, Nicky D’Haene, Serge Nolet, Ari Ristimäki, Timo Väisänen, Caroline Chapusot, Afsaneh Soruri, Tina Unger, Johanna Wecgowiec, Michele Biscuola, Milo Frattini, Anna Long, Paulo V Campregher, Xavier Matias-Guiu

**Affiliations:** 1 Departments of Pathology and Molecular Genetics, Hospital U Arnau de Vilanova and Hospital U de Bellvitge, University of Lleida, IRBLLEIDA, IDIBELL, CIBERONC, Av. Alcalde Rovira Roure, 80 25198 Lleida, Spain; 2 Department of Pathology, Acıbadem Mehmet Ali Aydınlar University, Istanbul, Turkey; 3 Molecular Pathology Laboratory, Department of Pathology, afs. 134, Hvidovre Hospital, Hvidovre, Denmark; 4 Molecular Pathology Diagnostic Service, University Hospitals Birmingham NHS Foundation Trust, Birmingham, UK; 5 Städtisches Klinikum Karlsruhe gGmbH, Institut für Pathologie, Karlsruhe, Germany; 6 Ophthalmic Pathology Laboratory Histopathology, Royal Hallamshire Hospital, Glossop Road, Sheffield, UK; 7 Department of Pathology, University Medical Center Utrecht, Utrecht, The Netherlands; 8 Molecular Pathology Centre, Jewish General Hospital-McGill University, Montreal, Quebec Canada; 9 Laboratoire de Biopathologie, Unité INSERM UMR-S 1250, CHU Reims, Reims, France; 10 Department of Clinical Pathology and Cytology, Karolinska University Hospital, Stockholm, Sweden; 11 Molecular Pathology Unit, Department of Pathology, Oslo University Hospital, Oslo, Norway; 12 Department of Pathology, Forensic Medicine and Cytology, University Hospital Split, Split, Croatia; 13 Histopathology Department, Barking, Havering and Redbridge University Hospitals NHS Trust, Queen’s Hospital, Romford, UK; 14 Bioptická laboratoř s.r.o., Laboratory of Molecular Genetics, Plzeň, Czech Republic; 15 ÚBLG FN Motol, Praha, Czech Republic; 16 LF UK, Plzeň, Czech Republic; 17 Institute of Pathology, Dessau, Germany; 18 Department of Pathology, Shaukat Khanum Memorial Cancer Hospital & Research Centre, Johr Town, Lahore, Pakistan; 19 Pathologisch-Bakteriologisches Institut Kaiser-Franz-Josef-Spital, Vienna, Austria; 20 Zentrum für Pathologie Kempten-Allgäu (MVZ), Kempten, Germany; 21 Department of Pathology, Hospital Universitari Vall d’Hebron, Barcelona, Spain; 22 Spanish Biomedical Research Network Centre in Oncology (CIBERONC), Madrid, Spain; 23 Anatomia Patológica Rede D’Or, São Paulo, SP Brazil; 24 Department of Pathology, Hadassah Medical Center, Jerusalem, Israel; 25 Department of Pathologic Anatomy, Jessenius Faculty of Medicine, Comenius University in Bratislava, Martin, Slovak Republic; 26 Martin’s Biopsy Center Ltd., Martin, Slovak Republic; 27 Hong Kong Molecular Pathology Diagnostic Centre, Hong Kong Special Administrative Region of the People’s Republic of China, Hong Kong, People’s Republic of China; 28 Genomics and Molecular Biology Group, International Research Center/CIPE, A. C. Camargo Cancer Center, São Paulo, SP Brazil; 29 GenoMed, Diagnósticos de Medicina Molecular, SA, Lisbon, Portugal; 30 Molecular Oncology Research Center, Barretos Cancer Hospital, Barretos, SP Brazil; 31 Life and Health Sciences Research Institute (ICVS), School of Medicine, University of Minho, Braga, Portugal; 32 ICVS/3B’s - PT Government Associate Laboratory, Braga/Guimarães, Portugal; 33 Pathology, Peter MacCallum Cancer Centre and University of Melbourne, Vic, Australia; 34 Surgical Pathology Unit, Department of Medicine (DIMED), University of Padua, Padua, Italy; 35 Department of Pathology, Hospices Civils de Lyon, Université Lyon 1, Bron, France & Cypath, Villeurbanne, France; 36 Institute of Pathology, University Hospital Cologne, Cologne, Germany; 37 Nuffield Department of Surgical Sciences, University of Oxford, Oxford, UK; 38 Department of Pathology, University of Cambridge, Cambridge, UK; 39 Department of Pathology, Tan Tock Seng Hospital, Novena, Republic of Singapore; 40 Department of Pathology, Erasme Hospital, Université Libre de Bruxelles (ULB), Brussels, Belgium; 41 Département de Pathologie et Biologie Cellulaire, Université de Montréal, Montreal, Québec Canada; 42 Department of Pathology, Research Programs Unit and HUSLAB, University of Helsinki and Helsinki University Hospital, Helsinki, Finland; 43 Oulu University Hospital and Department of Pathology, Cancer and Translational Medicine Research Unit, University of Oulu, Oulu, Finland; 44 Platform of Somatic Oncology of Burgundy, CHU, Dijon, France; 45 Institut für Pathologie und Molekularpathologie, Pforzheim, Germany; 46 Institut für Pathologie, University of Leipzig, Leipzig, Germany; 47 Institut für Pathologie, Evangelisches Krankenhaus BETHESDA Zu Duisburg GmbH, Duisburg, Germany; 48 Department of Pathology, Molecular Pathology Laboratory, Hospital Universitario Virgen del Rocío-IBIS, Seville, Spain; 49 Laboratory of Molecular Pathology, Institute of Pathology, Locarno, Switzerland; 50 Cellular Pathology, Newcastle Upon Tyne Hospitals NHS Foundation Trust, Newcastle upon Tyne, UK; 51 Hospital Israelita Albert Einstein, São Paulo, Brazil

**Keywords:** Microsatellite instability, Idylla™ MSI assay, Colorectal cancer, Multi-center study, FFPE clinical tissue samples

## Abstract

Microsatellite instability (MSI) is present in 15–20% of primary colorectal cancers. MSI status is assessed to detect Lynch syndrome, guide adjuvant chemotherapy, determine prognosis, and use as a companion test for checkpoint blockade inhibitors. Traditionally, MSI status is determined by immunohistochemistry or molecular methods. The Idylla™ MSI Assay is a fully automated molecular method (including automated result interpretation), using seven novel MSI biomarkers (*ACVR2A*, *BTBD7*, *DIDO1*, *MRE11*, *RYR3*, *SEC31A*, *SULF2*) and not requiring matched normal tissue. In this real-world global study, 44 clinical centers performed Idylla™ testing on a total of 1301 archived colorectal cancer formalin-fixed, paraffin-embedded (FFPE) tissue sections and compared Idylla™ results against available results from routine diagnostic testing in those sites. MSI mutations detected with the Idylla™ MSI Assay were equally distributed over the seven biomarkers, and 84.48% of the MSI-high samples had ≥ 5 mutated biomarkers, while 98.25% of the microsatellite-stable samples had zero mutated biomarkers. The concordance level between the Idylla™ MSI Assay and immunohistochemistry was 96.39% (988/1025); 17/37 discordant samples were found to be concordant when a third method was used. Compared with routine molecular methods, the concordance level was 98.01% (789/805); third-method analysis found concordance for 8/16 discordant samples. The failure rate of the Idylla™ MSI Assay (0.23%; 3/1301) was lower than that of referenced immunohistochemistry (4.37%; 47/1075) or molecular assays (0.86%; 7/812). In conclusion, lower failure rates and high concordance levels were found between the Idylla™ MSI Assay and routine tests.

## Introduction

Colorectal cancer (CRC) is a serious health problem in western countries. In 2018 in Europe, CRC was the second most commonly diagnosed malignancy (500,000 cases) and also the second leading cause of cancer death (243,000 deaths), with a total of 4.51 million new cancer cases overall [1, 2]. The general population has a lifetime risk for developing CRC of about 5% [3, 4].

Environmental and hereditary factors contribute to its development, as demonstrated by the accumulation of mutations in oncogenes, tumor suppression, and mismatch repair deficiency. CRCs comprise a group of molecularly heterogeneous tumors that are characterized by a range of genomic and epigenomic alterations. A significant proportion of colorectal carcinomas show chromosomal instability and follow the classical morphological progression sequence in the adenoma/carcinoma pathway genes [5, 6].

Microsatellite instability (MSI) was initially noted in cancers of patients with Lynch syndrome, often called hereditary non-polyposis colon cancer syndrome (HNPCC), but also in some sporadic colon cancers [7–10]. Microsatellite instability high (MSI-H) is a phenomenon present in approximately 15 to 20% of primary CRCs and is characterized by mutation or methylation of mismatch repair (*MMR*) genes [11]. CRC patients from HNPCC kindred have an inherited germline mutation in either *MLH1*, *MSH2*, *MSH6*, *PMS2*, or *EPCAM*. This germline alteration is combined with a somatic alteration in the contralateral allele, fulfilling Knudson’s two hits [12]. The MSI-associated MMR deficiency leads to the accumulation of myriads of mutations in coding and non-coding DNA sequences, generating instability in the microsatellite regions, which expand or contract with the insertion or deletion of repetition units, characteristic of the hypermutator phenotype. Approximately 20 to 25% of MSI-H CRCs represent HNPCC-related tumors, while the remaining 75 to 80% corresponds to sporadic CRCs [13]. Thus, MSI-H is a critical marker for the diagnosis of HNPCC.

It has been suggested that identification of MSI in CRC is important for assessment of prognosis and treatment stratification. Fluorouracil (5-FU) is a component of the standard treatment for patients with stage II CRC. There is evidence in the literature suggesting that MMR deficiency is associated with 5-FU resistance in CRC cells. Thus, MSI testing is helpful in the clinical assessment and management of CRC patients because MSI-H tumors are associated with a favorable prognosis after surgical resection and do not have improvement in survival with the addition of adjuvant 5-FU therapy [14].

MSI-H tumors may be targets for immunotherapeutic treatments. Immune checkpoint inhibitors have moved the field of immuno-oncology to the forefront of cancer treatment, and immune checkpoint blockade therapies have been FDA-approved for the treatment of a broad range of tumor types, including CRC. The presence of MSI is now established as a biomarker for response to immunotherapy; Brahmer suggested that MSI-H tumors are hypermutated and express numerous neoantigens caused by mutations and a high number of frameshifts that induce immune responses by tumor-infiltrating lymphocytes (TILs) [15].

Several techniques can be used for MSI testing. Microsatellite insertions and deletions (indels) can be demonstrated by extracting DNA from formalin-fixed, paraffin-embedded (FFPE) normal and CRC tumor tissue and subsequent amplification of specific microsatellite sequences by polymerase chain reaction (PCR) and fragment size analysis [16]. Bacher identified an optimal set of markers that provided maximal sensitivity and specificity for MSI-H tumors and incorporated them into a multiplex fluorescent assay for a simple, rapid, and accurate detection of MSI-H tumor phenotype. [17] The resulting Promega assay for MSI testing included five nearly monomorphic mononucleotide repeat markers: *BAT-25*, *BAT-26*, *NR-21*, *NR-24*, and *MONO-27*. All of them are mononucleotide repeat markers previously reported to have greater sensitivity and specificity for MMR deficiency than dinucleotide markers. An updated version of the assay includes the five mononucleotide markers and two highly polymorphic pentanucleotide repeat markers (Penta C and Penta D).

Immunohistochemistry (IHC) shows a 95% sensitivity for DNA MMR deficiency, and this technique consists of detecting the expression of proteins from the major DNA *MMR* genes (*MSH2* and *MLH1*) and from the minor DNA *MMR* genes (*MSH6* and *PMS2*). It is important to keep in mind that loss of expression of any of the MMR proteins can also be due to bi-allelic somatic inactivation.

Both IHC and the previously mentioned MSI DNA tests are sensitive and specific, but there is room for improvement. The reported sensitivity of MSI DNA tests is 89% for *MLH1*/*MSH2* and 77% for *MSH6*, while the reported sensitivity of IHC is 77 to 83%. [20] Concordance between both methods is over 92%. [17–20]

The updated relevance of MSI in CRC, not only by identifying HNPCC patients that present as sporadic CRC but also in prognosis and in treatment decisions regarding adjuvant chemotherapy (5-FU) and immunotherapy, justifies efforts to improve the currently available techniques. The search for optimal methods for MSI testing, with simpler workflow and less requirements regarding tumor tissue availability is an additional driver for this research.

Whole-genome and whole-exome sequencing of MMR-deficient tumors (endometrial cancer and CRC) and of normal cells led to the identification of 59 new biomarkers being indicative for MSI status [21]. Based on this panel, the Idylla™ MSI assay was developed, which contains seven novel MSI biomarkers selected on their stability over different cancer types and ethnicities, and showing high diagnostic performance: *ACVR2A*, *BTBD7*, *DIDO1*, *MRE11*, *RYR3*, *SEC31A*, and *SULF2* [22]. When tested on smaller sets of CRC FFPE tissue samples, the Idylla™ MSI assay showed concordance rates of > 97.5% with previous routine IHC and molecular results [22–24]. The current study describes a multi-center evaluation (44 centers) of the performance of the Idylla™ MSI assay in comparison with IHC or with molecular tests including the Bethesda/Promega MSI Analysis System on 1301 archival CRC FFPE tissue sections.

## Materials and methods

### Tissue sample collection

For this study, archived clinical FFPE tissue material of 1301 CRC patients was selected by the participating clinical centers. The samples were obtained from 44 clinical centers around the globe (Table 1).

**Table 1:** Overview of the 44 clinical centers participating in the multi-center study

Institution	Location	Number of samples tested	IHC panel	Molecular method panel
Department of Pathology, Hospital Universitari Vall d’Hebron	Barcelona, Spain	30	MLH1, MSH2, PMS2, MSH6	*BAT-25, BAT-26, NR-21, NR-24, MONO-27*
Barretos Cancer Hospital	Barretos, Brazil	26	MLH1, MSH2, PMS2, MSH6	*BAT-25, BAT-26, NR-21, NR-24, NR-27, HSP110*
University Hospital Birmingham	Birmingham, UK	28	MLH1, MSH2, PMS2, MSH6	NA
Hôpital Erasme Service d’Anatomie Pathologique	Brussels, Belgium	30	MLH1, MSH2, PMS2, MSH6	*BAT-25, BAT-26, NR-21, NR-24, MONO-27*
Addenbrooke’s Hospital *AND* Department of Cellular Pathology (Oxford University Hospitals NHS Foundation Trust)	Cambridge, UK and Oxford, UK	30	NA	*BAT-25, BAT-26, NR-21, NR-24, MONO-27*
Institute of Pathology, University Hospital Cologne	Cologne, Germany	30	MLH1, MSH2, PMS2, MSH6	*BAT-25, BAT-26, NR-21, NR-22, NR-27 OR BAT-25, BAT-26, D2S123, D5S346, D17S250 OR BAT-25, BAT-26, BAT-40, NR-21, NR-22, NR-24, NR-27, D2S123, D5S346, D17S250, D10S197, D18S58, D13S153, MYCL1*
Hvidovre Hospital	Copenhagen, Denmark	32	MLH1, MSH2, PMS2, MSH6	NA
Städtisches Klinikum Dessau, Institut für Pathologie, Abteilung für Molekularpathologie	Dessau, Germany	30	MLH1, MSH2, PMS2, MSH6	*BAT-25, BAT-26, NR-21, NR-24, MONO-27*
Platform of Somatic Oncology of Burgundy, CHU de Dijon	Dijon, France	30	MLH1, MSH2, PMS2, MSH6	*BAT-25, BAT-26, NR-21, NR-24, MONO-27*
Ev. Krankenhaus Bethesda, Institut für Pathologie	Duisburg, Germany	30	MLH1, MSH2, PMS2, MSH6	*BAT-25, BAT-26, NR-21, NR-24, MONO-27*
Pathology, HUSLAB, Helsinki University Hospital	Helsinki, Finland	30	MLH1, MSH2, PMS2, MSH6	NA
Hong Kong Molecular Pathology Diagnostic Centre	Hong Kong Special Administrative Region of the People’s Republic of China, China	30	MLH1, MSH2, PMS2, MSH6	*BAT-25, BAT-26, NR-21, NR-24, MONO-27*
Acibadem Pathology	İstanbul, Turkey	30	MLH1, MSH2, PMS2, MSH6	*BAT-25, BAT-26, NR-21, NR-24, MONO-27 OR BAT-25, BAT-26, NR-21, NR-22, NR-24*
Hadassah Ein Kerem Medical Center	Jerusalem, Israel	30	MLH1, MSH2, PMS2, MSH6	*BAT-25, BAT-26, NR-21, NR-24, MONO-27*
Städtisches Klinikum Karlsruhe gGmbH, Pathologisches Institut	Karlsruhe, Germany	30	MLH1, MSH2, PMS2, MSH6	*BAT-25, BAT-26, D2S123, D5S346, D17S250*
Zentrum für Pathologie Kempten - Allgäu	Kempten, Germany	30	MLH1, MSH2, PMS2, MSH6	*BAT-25, BAT-26, NR-21, NR-22, NR-24 OR BAT-25, BAT-26, NR-21, NR-24, NR-27*
Shaukat Khanum Cancer Hospital and Research Centre	Lahore, Pakistan	27	MLH1, MSH2, PMS2, MSH6	NA
Institut für Pathologie, Universitätsklinikum Leipzig	Leipzig, Germany	32	MLH1, MSH2, PMS2, MSH6	*BAT-25, BAT-26, D2S123, D5S346, D17S250*
GenoMed - Diagnósticos de Medicina Molecular, SA	Lisbon, Portugal	30	MLH1, MSH2, MSH6	*BAT-25, BAT-26, NR-21, NR-24, MONO-27*
Hospital Universitari Arnau de Vilanova	Lleida, Spain	31	MLH1, MSH2, PMS2, MSH6	*BAT-25, BAT-26, NR-21, NR-24, MONO-27*
Istituto Cantonale di Patologia	Locarno, Switzerland	30	MLH1, MSH2, PMS2, MSH6	*BAT-25, BAT-26, D2S123, D5S346, D17S250*
CHU Lyon Est	Lyon, France	29	MLH1, MSH2, PMS2, MSH6	*BAT-25, BAT-26, NR-21, NR-24, MONO-27*
MBC, Ltd.	Martin, Slovak Republic	34	MLH1, MSH2, PMS2, MSH6	*BAT-25, BAT-26, NR-21, NR-24, MONO-27*
Peter MacCallum Cancer Centre	Melbourne, Australia	27	NA	*BAT-25, BAT-26, NR-21, NR-22, D2S123, D5S346, D17S250, CAT25*
CHUM	Montréal, Canada	30	MLH1, MSH2, PMS2, MSH6	*BAT-25, BAT-26, NR-21, NR-22, NR-24, MONO-27*
Jewish General Hospital (LDI)	Montréal, Canada	30	MLH1, MSH2, PMS2, MSH6	NA
Cellular Pathology, RVI	Newcastle upon Tyne, UK	30	MLH1, MSH2, PMS2, MSH6	*BAT-25, BAT-26, NR-21, NR-24, MONO-27*
Olso University Hospital	Oslo, Norway	28	NA	*BAT-25, BAT-26, BAT-40, D2S123, D5S346, D18S69*
Oulu University Hospital, Department of Pathology	Oulu, Finland	30	MLH1, MSH2, PMS2, MSH6	NA
Surgical Pathology Unit, Department of Medicine (DIMED) - University of Padua	Padua, Italy	30	MLH1, MSH2, PMS2, MSH6	*BAT-25, BAT-26, BAT-40, NR-21, NR-24, D2S123, D5S346, D17S250, D18S58, TGFbetaRII*
Institut für Pathologie und Molekularpathologie Pforzheim	Pforzheim, Germany	23	MLH1, MSH2, PMS2, MSH6	NA
Bioptická Laboratoř s.r.o.	Pilsen, Czech Republic	30	MLH1, MSH2, PMS2, MSH6	*BAT-25, BAT-26, NR-21, NR-24, MONO-27*
CHU Reims, Laboratoire de Biopathologie HMB, Hôpital Maison Blanche	Reims, France	32	MLH1, MSH2, PMS2, MSH6	*BAT-25, BAT-26, NR-21, NR-24, MONO-27*
Anatomia patológica Rede D’Or	Rio de Janeiro, Brazil	30	MLH1, MSH2, PMS2, MSH6	NA
BHRUT - Queen’s Hospital	Romford, UK	30	MLH1, MSH2, PMS2, MSH6	NA
AC Camargo Cancer Center	São Paulo, Brazil	29	MLH1, MSH2, PMS2, MSH6	*BAT-25, BAT-26, NR-21, NR-24, MONO-27*
Instituto do Cancer do Estado de São Paulo	São Paulo, Brazil	30	MLH1, MSH2, PMS2, MSH6	NA
Molecular Pathology Lab, Pathology Department, Virgen del Rocío Hospital	Seville, Spain	30	MLH1, MSH2, PMS2, MSH6	*BAT-25, BAT-26, NR-21, NR-24, MONO-27*
STH Histopathology	Sheffield, UK	30	MLH1, MSH2, PMS2, MSH6	NA
Department of Pathology, Tan Tock Seng Hospital	Singapore, Republic of Singapore	30	MLH1, MSH2, PMS2, MSH6	*BAT-25, BAT-26, NR-21, NR-24, MONO-27*
University Hospital Split	Split, Croatia	30	MLH1, MSH2, PMS2, MSH6	NA
Department of Clinical Pathology and Cytology, Karolinska University Hospital, Stockholm, Sweden	Stockholm, Sweden	30	MLH1, MSH2, PMS2, MSH6	*BAT-25, BAT-26, NR-21, NR-24, MONO-27*
University Medical Center Utrecht	Utrecht, The Netherlands	30	MLH1, MSH2, PMS2, MSH6	*BAT-25, BAT-26, BAT-40, D2S123, D5S346, D17S250*
Pathologisch-Bakteriologisches Institut, KFJ-Spital	Wien, Austria	23	MLH1, MSH2, PMS2, MSH6	*BAT-25, BAT-26, NR-21, NR-24, MONO-27*

*NA*, not assessed

The use of the patient samples was approved by the respective local Ethics Committees and was in compliance with the Declaration of Helsinki. The participating centers received proper training to perform the Idylla™ MSI assay.

The Idylla™ MSI assay was performed on slides/slices from the same block of archived clinical FFPE tissue material used previously for testing with routine reference methods, and slices/slides were taken as close as possible to the sample used for these routine reference methods.

### Idylla™ MSI assay

The Idylla™ MSI assay, performed on the Idylla™ System, is intended for the qualitative detection of a panel of seven monomorphic homopolymer biomarkers for identification of microsatellite instability in human cancer, resulting in identification of the MSI status of the sample. These novel MSI markers used in the Idylla™ MSI assay are (with locus between brackets): *ACVR2A* (2q22.3-q23.1), *BTBD7* (14q32.12), *DIDO1* (20q13.33), *MRE11* (11q21), *RYR3* (15q13.3-q14), *SEC31A* (4q21.22), and *SULF2* (20q13.12). They were selected to be short and monomorphic, in order to be compatible with PCR detection by means of probes rather than analysis by means of capillary electrophoresis. The Idylla™ MSI assay uses FFPE material from human cancer tissue, which is directly loaded in the cartridge.

The tissue area of the FFPE specimen should be between 50 and 600 mm^2^ when 5-μm FFPE tissue sections are used and between 25 and 300 mm^2^ when using 10-μm FFPE tissue sections; up to five FFPE tissue sections can be used to meet this requirement. If a specimen contains less than 20% neoplastic cells, macro-dissection has to be performed. The Idylla™ MSI assay automates the entire process from FFPE sample preparation to reporting of MSI status, including liberation of nucleic acids from FFPE material, PCR amplification, and analysis by high-resolution melting detection. The total turnaround time of the Idylla™ MSI assay is less than 150 min.

According to the manufacturer’s assay instructions, the MSI status of the sample can be determined with high confidence if at least five valid marker-specific fluorescence profiles could be fully analyzed (otherwise the MSI status will be called “invalid”). At least two mutant markers will result in a status being “MSI-H” (microsatellite instability high), otherwise the status will be scored as “MSS” (microsatellite stable).

### IHC and molecular routine reference methods

IHC analysis of the expression of the marker genes *MLH1*, *MSH2*, MSH6, and *PMS2* in FFPE tissue material was performed using routine standard protocols and equipment, including commercial antibodies from Ventana (Roche Diagnostics, Rotkreuz, Switzerland) and Dako (Agilent, Santa Clara, CA), and systems from Ventana and Leica Biosystems (Wetzlar, Germany). There was no central re-review and that the results of these routine tests were reviewed in retrospect.

Investigation of colorectal FFPE tissue material with the PCR-based Promega MSI Analysis System v1.2 (RUO), which analyzes the five MSI markers from the revised Bethesda panel, i.e., *NR-21*, *NR-24*, *BAT-25*, *BAT-26*, and *MONO-27*, was performed according to the procedures implemented in every individual lab (Table 1). Alternatively, PCR analysis was performed on customized molecular MSI panels including markers from the following list: *NR-21*, *NR-22*, *NR-24*, *NR-27*, *BAT-25*, *BAT-26*, *BAT-40*, *D2S123*, *D5S346*, *D10S197*, *D13S153*, *D17S250*, *D18S58*, *D18S69*, *CAT25*, *HSP110*, *TGFbetaRII*, and *MYCL1*.

For the molecular reference methods, criteria for defining MSI-H and MSS were according to the manufacturer’s assay instructions and each laboratory procedure. For the IHC reference methods, variability has been detected, as some centers defined the loss of one protein marker as being sufficient to call it deficient MMR, whereas other centers defined the loss of a paired protein marker. In this the study, the site-specific standard laboratory procedure was followed to present a real-life data cohort.

### Statistical analysis

The agreement between the Idylla™ MSI assay and the comparator methods (IHC or PCR-based assays on MSI panels) was evaluated based on point estimates for Overall, Positive, and Negative Percent Diagnostic agreement together with 95% one-sided Wilson-score confidence intervals.

## Results

### FFPE tissue samples

The MSI status of archived clinical FFPE tissue sections originating from 1301 patients with CRC was determined using the Idylla™ MSI assay at 44 centers. To increase the percentage of the tumor area for samples with low tumor cellularity to ≥ 20% as required by the instructions for use of the Idylla™ MSI assay, macro-dissection was performed in 552 cases. The sample characteristics are summarized in Table 2.

**Table 2:** Characteristics of the 1301 study samples

Characteristic	Number of samples
Tissue origin	
Primary	969
Metastatic	48
NA	284
Slice thickness (μm)	
3	30*
4	23*
5	671
8	1
10	553
NA	23
Number of slices	
1	939
2	164
3	120
4	52
5	19
6	3*
7	1*
8	1*
11	1*
12	1*
NA	0
% Tumor cells (after macro-dissection)	
< 10	4*
10– < 20	20*
20– < 30	76
30– < 40	153
40– < 50	135
50– < 60	158
60– < 70	165
70– < 80	146
80– < 90	113
90–100	84
NA	247

*NA*, not assessed

*Values not according to the specifications of the Idylla™ MSI assay instructions; however, for all these samples, Idylla™ MSI assay results were found concordant with results of previous routine reference methods

### Idylla™ MSI assay on archived clinical samples

All 1301 samples had previously been tested with at least one routine reference method, and 586 of the samples had been tested with both IHC and a molecular reference method. In total, 1075 samples had been tested before with IHC and 812 with molecular methods. Of the 812 samples tested with molecular methods, 101 had been tested with the original Bethesda panel, 525 with the revised Bethesda panel, and 186 against a range of other microsatellite biomarker panels (Table 1).

Of the 1301 samples tested with the Idylla™ MSI assay, 612 were found to be MSI-H and 686 to be MSS, while for three samples, the result was invalid. The majority (84.48%) of the MSI-H samples had five or more mutated biomarkers, and the vast majority (98.25%) of the MSS samples had zero mutated biomarkers (Table 3). Of the biomarkers tested by the Idylla™ MSI assay, *MRE11* gave most of the invalid results, while *DIDO1* did not result into any invalids; mutations were rather equally distributed over all of the seven assessed biomarkers (Table 4).

**Table 3:** Number of Idylla™ MSI Assay “mutant” calls

MSI status	Number of samples	Number of mutant markers	Number of samples	% of MSI-H
MSS	686	0	674	NA
		1	12	NA
MSI-H	612	2	15	2.45
		3	28	4.58
		4	52	8.50
		5	156	25.49
		6	226	36.93
		7	135	22.06
Invalid	3	NA	NA	NA
Total	1301			

*NA*, not applicable

**Table 4:** Idylla™ MSI assay calls per biomarker

	*ACVR2A*	*BTBD7*	*DIDO1*	*MRE11*	*RYR3*	*SEC31A*	*SULF2*
Overall							
Mutant	579	514	576	505	411	373	457
Wild-type	718	782	725	780	887	926	840
Invalid	4	5	0	16	3	2	4
Total	1301	1301	1301	1301	1301	1301	1301
MSI-H samples							
Mutant	575	513	570	504	411	373	457
Wild-type	37	99	42	107	201	239	154
Invalid	0	0	0	1	0	0	1
Total	612	612	612	612	612	612	612
MSS samples							
Mutant	4	1	6	1	0	0	0
Wild-type	680	683	680	672	685	686	685
Invalid	2	2	0	13	1	0	1
Total	686	686	686	686	686	686	686
Invalid samples							
Mutant	0	0	0	0	0	0	0
Wild-type	1	0	3	1	1	1	1
Invalid	2	3	0	2	2	2	2
Total	3	3	3	3	3	3	3

The failure rate of the Idylla™ MSI assay was 0.23% (3/1.301), while the reference methods had higher failure rates of 4.37% (47/1075) for IHC and 0.86% (7/812) for the routine molecular methods. Routine method failure rates might however be an underestimation as the current analysis was done retrospectively on samples with known routine results.

To investigate concordance levels, the results of the Idylla™ MSI assay were compared with the results of the routine reference methods, i.e., IHC or molecular MSI panels, performed before on slides/slices of the same FFPE block. According to the protocol, the FFPE slides/slices used for the Idylla™ MSI assay had to be taken as close as possible (within the block) to the slides/slices used to generate the reference result. Although this was not always the case, sections were close in the vast majority of cases. Also, a hematoxylin and eosin (H&E) staining to confirm of the presence of tumor tissue in the sample was not done at all sites.

### Concordance of Idylla™ MSI assay results with routine IHC results

Compared to IHC, the results of 37 of the 1025 samples tested (valid calls only) with the Idylla™ MSI Assay on the same FFPE block were reported to be discordant; i.e., a concordance of 96.39% (CI: 95.06–97.37%) was obtained (Table 5).

**Table 5:** Comparison between the results of the Idylla™ MSI assay and the results of routine IHC assays or of routine molecular methods

		IHC				
		dMMR	pMMR	Invalid	Doubtful^a^	Total
Idylla™	MSI-H	501	12	1	10	524
	MSS	25	487	6	30	548
	Invalid	2	1	0	0	3
	Total	528	500	7	40	1075
Idylla™ Performance	Positive agreement	501/526 = 95.24% (CI: 93.08–96.76%)				
	Negative agreement	487/499 = 97.59% (CI: 95.84–98.62%)				
	Overall agreement	988/1025 = 96.39% (CI: 95.06–97.37%)				
		Molecular methods				
		MSI-H	MSS	Invalid	Doubtful^a^	Total
Idylla	MSI-H	381	4	0	2	387
	MSS	12	408	1	2	423
	Invalid	0	0	2	0	2
	Total	393	412	3	4	812
Idylla™ Performance	Positive agreement	381/393 = 96.95% (CI: 94.74–98.24%)				
	Negative agreement	408/412 = 99.03% (CI: 97.53–99.62%)				
	Overall agreement	789/805 = 98.01% (CI: 96.80–98.77%)				

^a^Doubtful results have been reported by the involved clinical site as doubtful based on their site-specific evaluation criteria, which were not standardized across sites

For 21 of these 37 discordant samples, results of molecular methods were also available, and in 14 cases, these results were concordant with the results of the Idylla™ MSI Assay (i.e., 5 MSI-H and 9 MSS results). Of the 7 samples having discordant results between the Idylla™ MSI assay and both routine reference methods, 2 samples were retested with the Idylla™ MSI assay, and this retest confirmed the results of the reference methods (i.e., deficient mismatch repair (dMMR) and MSI-H).

Of the remaining 16 of the 37 discordant samples, which were only tested with IHC as a reference, a retest result on consecutive slides/slices with the Idylla™ MSI assay was available for 1 sample and found to be concordant with IHC (i.e., MSI-H/dMMR).

For the 47 of the 1025 samples tested that had invalid or doubtful IHC calls, molecular method results for the same tissue block were available in 45 cases, of which 42 were found to be concordant with the Idylla™ MSI Assay (i.e., 9 MSI-H/dMMR and 33 MSS/proficient mismatch repair (pMMR)).

### Concordance of Idylla™ MSI assay results with routine molecular method results

Compared to molecular methods, the results of 16 of the 805 samples tested (valid calls only) with the Idylla™ MSI assay on the same FFPE block were reported to be discordant; i.e., a concordance of 98.01% (CI: 96.80–98.77%) was obtained (Table 5).

For 12 of these 16 discordant samples, IHC had also been performed previously, and in 4 cases, these results were found to be concordant with the Idylla™ MSI assay (i.e., 4 MSS); in these 4 cases, the marker panel used in the molecular reference method encompassed dinucleotide repeats. Of the 8 samples having discordant results between the Idylla™ MSI assay and both routine reference methods, 2 samples were retested with the Idylla™ MSI assay confirming reference results as described above.

Of the remaining 4 of the 16 discordant samples, which were only tested with molecular methods as a reference, a retest result on consecutive slides/slices with the Idylla™ MSI assay was available for 2 samples. For 1 of these 2 samples, the retest result was concordant with the result of the molecular method (i.e., MSI-H); improper cutting of the tumor from the original block is suspected to have caused the discordant MSS result for the Idylla™ MSI assay. For the second sample, the retest still detected an MSI-H status discordant with the MSS status determined with the Promega MSI Analysis System; however, an additional IHC analysis of this sample found dMMR, which is concordant with the Idylla™ MSI assay result.

For the 7 samples with invalid or doubtful results by a molecular method, IHC results were available in 3 cases, which were found to be concordant with the Idylla™ MSI assay results (i.e., 1 MSI-H/dMMR and 2 MSS/pMMR).

## Discussion

In the current article, we report the results of a multi-center study of the Idylla™ MSI assay on archival FFPE CRC tumor tissue from different countries around the globe, and hence different ethnicities, to assess concordance with previously obtained results from both IHC and molecular tests. A total of 1301 samples were analyzed in 44 independent centers selected from 25 different countries. A total of 612 clinical samples were classified by the Idylla™ MSI assay as MSI-H and 686 samples as MSS, while only 3 cases were invalid. The samples had been tested before either by IHC (1075 cases) and/or by molecular methods (812 cases).

Discordant results between the Idylla™ MSI assay and IHC were detected in 37 of 1025 cases, resulting in a concordance level of 96.39%. Discordance between the Idylla™ MSI Assay and other molecular methods, including the Promega MSI Analysis System, was found in 16 of the 805 cases, which represents a 98.01% concordance. Of the 1301 samples, 586 were tested with both IHC and molecular methods, which enabled comparison of the results of three methods. As a result, of the 37 samples discordant between the Idylla™ MSI assay and IHC, 14 were concordant between the Idylla™ MSI assay and molecular methods, and conversely, of the 16 samples discordant between the Idylla™ MSI assay and molecular methods, 4 were concordant between the Idylla™ MSI Assay and IHC. Due to a restricted amount of archived sample tissue available, only 5 of the discordant results were retested with the Idylla™ MSI assay, and no further analysis with other methods was performed. The excellent concordance levels found are in line with previously published levels for the Idylla™ MSI assay [22–25]. In these studies with smaller sample sets and more standardized routine reference methods, concordance levels of the Idylla™ MSI assay were 95.00–98.71% with IHC, 99.05–100.00% with molecular methods, and 99.05% with next-generation sequencing.

The mutations were found to be rather equally distributed over the seven biomarkers of the Idylla™ MSI assay. The vast majority of the MSS calls had no mutations in the seven biomarkers, while the majority of the MSI-H calls had at least five mutated biomarkers. Taking also into account the global multi-center, these findings not only underscore the high and consistent incidence of the seven assessed biomarkers in CRC but also their stability across different regions worldwide (excluding Africa and North America) and hence indirectly different ethnicities. Of the 12 MSS samples with only one mutated Idylla™ MSI biomarker, 5 had an MSI-H call when using the routine reference methods, indicative for a low Idylla™ false-negative rate. Moreover, of these 5 samples, 1 was reported to have < 5% tumor cells, which is below the minimal percentage stipulated in the Idylla™ MSI assay instructions for use, and for 3 of these samples, the exact neoplastic cell content was unknown. Therefore, too low levels of tumor cells in these samples may have been the reason why the Idylla™ MSI assay did only find one biomarker to be mutated.

It is highly important that assay instructions are followed, and for sure that the minimal amount of neoplastic cells (i.e., 20% for the Idylla™ MSI assay) has been obtained. As in the current study the Idylla™ MSI assay was performed on archival tissue samples, there is indeed a chance that some samples did not contain tumor tissues. In this respect, the protocol required sampling of slices/slides to happen as close as possible (within the block) to the slides/slices used to generate the reference result. This was not always the case and may have contributed to a number of MSS calls by the Idylla™ MSI assay that were not concordant with the MSI-H call of the reference method. In addition, an H&E confirmation of the presence of tumor tissue in the sample was absent in many cases.

Another disadvantage of our multi-center study of 44 centers in multiple countries is that the IHC and molecular routine reference methods used showed variability in marker panels, providers, protocols, interpretation, and scoring criteria, which may have influenced their outcome and as such the concordance rates found. However, this setup enabled testing and benchmarking of the Idylla™ MSI assay in a real-world setting, which was the main goal of this study. It also showed the stable performance of the Idylla™ MSI assay in different laboratory environments.

The failure rate of the Idylla™ MSI assay was only 0.23%, which is lower than that of IHC (4.37%) or of the routine molecular MSI tests (0.86%). However, the actual failure rates of the reference methods may be considerably higher, as the samples in the current study were retrospectively selected based on the availability of valid results from at least one reference method. The lower failure rate compared to other molecular methods might be explained by the shorter amplicons analyzed in the Idylla™ MSI assay (below 100 base pairs) compared with the Promega MSI Analysis System (150 base pairs or higher), which results in an improved performance of Idylla™ on bad-quality samples (highly fragmented DNA and/or low-input DNA samples). IHC needs visual result interpretation of immunostaining color patterns, which is done via site-dependent interpretation strategies and cutoff values that also introduce a subjective pathologist-dependent aspect. These issues may lead to higher numbers of invalid/doubtful results as we have observed in the current study, with 40 doubtful IHC results being recorded at seven sites. In contrast, the Idylla™ MSI assay’s software decision tree is fully automated, and therefore, results are not prone to subjective interpretation. A previous analysis of consecutive sections of 182 samples with three methodologies revealed a higher number of invalid results for the Promega MSI Analysis System (3.8%) and IHC (13.2%) compared with the prototype Idylla™ MSI assay (2.2%) [26]. As to interpretation strategies used for IHC results in the current study, the majority of the sites classified samples with at least one deficient marker as dMMR but two sites needed at least two deficient markers to do so. In 18 cases, not all four biomarkers (MLH1, MSH2, MSH6, PMS2) were tested with IHC, and of these, three results did not confirm the MSI-H status determined with the Idylla™ MSI assay.

Overall, IHC testing is highly specific and sensitive, with easy performance and cost effectiveness [20]. However, there are also limitations. For example, some mutations that are not detected by the antibodies used in IHC still result in expression of nonfunctional proteins. More importantly, poor pre-analytical conditions, particularly delayed or prolonged fixation, may be responsible for difficulties in interpretation in some cases. As a general rule, it is important to verify internal control staining in non-neoplastic cells to enable interpretation of the results. One additional advantage of IHC is the fact that the absence of expression of a specific MMR protein can direct germline testing to that specific gene.

The Idylla™ MSI assay differs from the Promega MSI Analysis System due to its seven alternative biomarkers. It has been suggested that the seven selected regions might show consistent wild-type profiles over different ethnicities, while for the Bethesda markers, there is an actual variation between different ethnicities [27]. Noteworthy, the current study was not specifically designed to address that issue. Therefore, to obtain a correct interpretation of the results, a comparison between a matched normal tissue and the tumor tissue profile for each patient needs to be performed when testing against the Bethesda panel, which is not the case for the Idylla™ MSI Assay that only requires tumor tissue testing, hence a simplified procedure. Further differences are that routine molecular methods have long turnaround times with cumbersome, lengthy workflows usually requiring batching of samples, and that capillary sequencing instrumentation is required for these methods. The Idylla™ MSI assay in contrast demonstrates great specificity in a highly automated simplified workflow compared to current methods, with reliable results within approximately 150 min. Although the study was not designed for this purpose, results may suggest that Idylla™ MSI assay may be less dependent on variations of pre-analytical conditions.

In summary, the Idylla™ MSI assay showed high concordances with IHC and molecular testing, with a simple workflow and short turnaround time. It required limited amount of tumor tissue (no matched normal tissue). This study has been performed with the *research use only* product, as this was the product available at that time. Currently, the company has launched the CE-marked labeled *in vitro diagnostic* (IVD) product.
